# Prognostic Implications of Physical Activity on Mortality from Ischaemic Heart Disease: Longitudinal Cohort Study Data

**DOI:** 10.3390/jcm12134218

**Published:** 2023-06-22

**Authors:** Dalia Luksiene, Vilma Jasiukaitiene, Ricardas Radisauskas, Abdonas Tamosiunas, Martin Bobak

**Affiliations:** 1 Laboratory of Population Studies of Institute of Cardiology, Medical Academy, Lithuanian University of Health Sciences, LT-44307 Kaunas, Lithuania; vilma.jasiukaitiene@lsmuni.lt (V.J.); ricardas.radisauskas@lsmuni.lt (R.R.); abdonas.tamosiunas@lsmuni.lt (A.T.); 2 Department of Epidemiology and Public Health, University College London, London WC1E 6BT, UK; m.bobak@ucl.ac.uk

**Keywords:** physical activity, mortality risk, ischaemic heart disease, longitudinal cohort study

## Abstract

Background: The prevalence of physical inactivity has been rising in many countries in recent years, adding to the burden of non-communicable diseases and affecting overall health worldwide. The aim of this study was to determine the comprehensive assessment of the prognostic value of physical activity in leisure time on mortality from ischemic heart disease (IHD) by gender separately for those respondents who were diagnosed with IHD and for those who were not diagnosed with IHD in their baseline health survey. Methods: In the baseline survey (2006–2008), 7100 men and women ages 45–72 were examined within the framework of the international study Health, Alcohol, and Psychosocial Factors in Eastern Europe (HAPIEE). A total of 6770 participants were available for statistical analysis (after excluding 330 respondents due to missing information on study variables). Physical activity was determined by leisure-time physical activities (hours/week). All participants in the baseline survey were followed up for IHD mortality events until 31 December 2018. Results: Using multivariate Cox regression analysis, it was found that moderate and higher levels of physical activity significantly reduced the risk of IHD mortality (HR = 0.54, *p* = 0.016 and HR = 0.60, *p* = 0.031, respectively) in men who were not diagnosed with IHD at baseline compared with physically inactive subjects. It was found that among men and women who were diagnosed with IHD at baseline, physical activity reduced the risk of mortality from IHD compared with those who were physically inactive (HR = 0.54, *p* = 0.021 and HR = 0.41, *p* = 0.025, respectively). Using mediation analysis, it was found that physical activity directly predicted statistically lower IHD mortality (*p* < 0.05) in men and women. Conclusion: High physical activity was a significant factor that directly predicted statistically lower IHD mortality in men, regardless of whether subjects had IHD at baseline or not. However, only moderate physical activity was a significant factor that directly predicted statistically lower IHD mortality in the women group with IHD at baseline.

## 1. Introduction

Despite earlier diagnosis and treatment of cardiovascular diseases (CVD), Lithuania has for many years had the highest mortality rate from CVDs among all causes of death. According to the data of the Center for Health Information of the Institute of Hygiene (Lithuania), in 2020, more than half of all deaths, i.e., 52.7%, were due to diseases of the circulatory system [1]. Thus, CVDs are a prevalent problem not only in Lithuania, where they rank first among all deaths, but also in Europe and worldwide. In the member countries of the European Society of Cardiology, an estimated 12.7 million new cases of CVD were diagnosed in 2019, and 113 million people have been diagnosed with CVD [2]. CVDs are also the most common cause of death in the 57 member countries of the European Society of Cardiology, with ischaemic heart disease (IHD) accounting for 45.0% of deaths in women and 39.0% of deaths in men [2].

Insufficient physical activity is one of the main risk factors for mortality worldwide. The prevalence of physical inactivity has been rising in many countries in recent years, adding to the burden of non-communicable diseases and affecting overall health worldwide [3,4]. Globally, 1 in 4 adults does not meet the recommended level of physical activity, and up to 5 million deaths per year could be prevented if the world’s population were more active [3]. People who are insufficiently active have about a 30.0% increased risk of death in comparison with people who are sufficiently active [3]. World Health Organization (WHO) guidelines demonstrate that compared to less active adult men and women, individuals who are more active have lower rates of all-cause mortality, coronary heart disease, stroke morbidity, high blood pressure, and metabolic syndrome [5]. The research data analysis of individual characteristics and physical activity domains in adults showed gender differences in physical activity levels in many studies [6,7].

The aim of this study was to determine the comprehensive assessment of the prognostic value of physical activity in leisure time on mortality from IHD by gender separately for those respondents who were diagnosed with IHD and for those who were not diagnosed with IHD in their baseline health survey in the context of other risk factors.

## 2. Materials and Methods

### 2.1. Study Design and Population

Health, Alcohol, and Psychosocial Factors in Eastern Europe (HAPIEE) study, a population-based urban cohort study conducted in Kaunas (Lithuania) [8]. Baseline data collection was carried out between 2006 and 2008. A sample of 7100 Kaunas men and women aged 45–72 years, stratified by sex and 5-year age groups, was randomly selected from the Kaunas population register. The response rate was 64.8%. The data from 6,770 respondents (3065 men and 3705 women) were used for the statistical analysis. The study protocol was approved by the Kaunas Regional Biomedical Research Ethics Committee (11 January 2005, Protocol No. 05/09). All participants signed an informed consent form.

### 2.2. Sociodemographic, Lifestyle Factors, and Psychological Well-Being

Sociodemographic factors were determined at the baseline survey using a standard questionnaire (age and education) [8]. Age was used as a continuous variable. Education was categorized into groups: 1. secondary education and lower; 2. college and higher education.

Lifestyle factors such as physical activity, nutrition, and smoking habits were evaluated using a standard questionnaire [8]. In order to assess the physical activity of the participants in their leisure time, 5 questions were asked. Physical activity was determined by the mean length of time spent per week during leisure time in autumn-winter and spring-summer seasons for walking, moderate and hard work, such as gardening, maintenance of the house, and other physical activities, such as engaging in sports, games, or hiking. Responses were based on the subjective opinions of the subjects. The calculation of physical activity hours per week showed statistically significant differences in means between men and women, so further analysis was calculated separately for men and women. Physical activity (hours/week) was ranked from minimum to maximum, dividing the respondents into three levels: inactive (tertile 1), moderately physically active (tertile 2), and higher physically active (tertile 3). Physical activity levels in the population aged 45–72 years are presented in Table 1.

Food frequency questionnaires (FFQs) were used to assess dietary habits. Subjects were asked about the frequency of consumption of 20 food groups (fresh or cooked vegetables, fruit, potatoes, cereals, meat, fish, dairy products, eggs, sweets, etc.). Subjects could choose one of six possible answer options: “rarely and never”, “2–3 times a month”, “once a week”, “2–3 times a week”, “4–6 times a week” or “daily”. Factor analysis was used to reduce the number of foods reported by the responders. Data on explanatory factor analysis were presented in our previous publication [9]. Factor analysis of the main dietary patterns revealed five-factor nutrition habits: consumption of fresh vegetables and fruit, consumption of sweets, consumption of porridge and cereals, consumption of meat, potatoes, and eggs, and consumption of chicken and fish. A dichotomous-dependent variable was constructed by dividing factor scores into two groups (1: more frequent than average consumption of a particular food group, 0: less frequent than average consumption).

Smoking status was classified as never smoking, former smoking, and current smoking. Current smokers were individuals who regularly smoked at least 1 cigarette per day.

Psychological well-being was assessed by a Control, Autonomy Self-realization, and Pleasure (CASP12) questionnaire [10]. For this measurement, participants were presented with a list of 12 statements that described their lives or how they felt. Their answers on a 4-point scale ranged from “never” to “often”, resulting in scores ranging from 12 to 48. The internal consistency of the CASP-12 scale was good (Cronbach’s alpha = 0.74). The psychological well-being scores of participants were classified into the category of a higher psychological well-being group if the scores were equal to or higher than the median (baseline survey: >40 in men and >38 in women). Participants with psychological well-being scores lower than the median were classified into the group with lower psychological well-being.

### 2.3. Objective Measurements

At the baseline survey, objective measurements (blood pressure, waist circumference), and biochemical analyses (high-density lipoprotein (HDL) cholesterol, fasting glucose, and triglycerides) were determined. Blood pressure (BP) was measured three times with an oscillometric device (Omron M5-1) after at least 5 min of rest in a seated position, and the mean values of systolic blood pressure (BP) and diastolic BP were taken. Fasting blood serum samples were analyzed at the WHO Regional Lipid Reference Center, Institute of Clinical and Experimental Medicine, Prague (Czech Republic). Lipid concentrations (triglycerides and high-density lipoprotein (HDL) cholesterol) in serum were measured on a Roche COBAS MIRA auto-analyser, using a conventional enzymatic method with reagents from Boehringer-Mannheim Diagnostics and Hoffmann-La Roche. The WHO Regional Lipid Reference Center was responsible for the quality control of biochemistry measures. The concentration of glucose in capillary blood was determined by an individual glucometer, “Glucotrend” [11].

Metabolic syndrome was diagnosed according to the National Cholesterol Education Program Adult Treatment Panel III (NCEP–ATP III) criteria [12]. Individuals with three or more of the five components were diagnosed with metabolic syndrome: (1) elevated arterial blood pressure (≥130/85 mmHg); (2) central obesity (waist circumference ≥ 102 cm for men, ≥88 cm for women); (3) impaired glucose regulation (fasting blood glucose ≥ 6.1 mmol/l); (4) elevated triglyceride levels (≥1.7 mmol/L); (5) low high-density lipoprotein (HDL) cholesterol concentration (<1.04 mmol/L in men, <1.3 mmol/L in women).

The epidemiological criteria for the identification of IHD at the baseline survey were, by priority: (1) exposure to myocardial infarction and/or ischaemic changes on the electrocardiogram (ECG), as assessed by Minnesota codes 1–1, 1–2, verified by information in medical documents (medical records, outpatient charts, etc.) [13]; (2) exertional angina pectoris identified using the G. Rose questionnaire [14]; (3) ischaemic ECG changes assessed using the following Minnesota codes: 1–3, 4–1, 4–2, 4–3, 5–1, 5–2, 5–3, 6–1, 6–2, 7–1, 8–3. Causes of death from IHD were coded according to the International Classification of Diseases (ICD)-10 codes (I20–I25).

The mortality from IHD among the study participants was monitored from the start of the primary health survey (2006) until 31 December 2018. The mean duration of follow-up and standard deviation were 10.55 ± 2.59 years.

### 2.4. Statistical Analysis

All data were analyzed using IBM SPSS (IBM Corp. Released 2020. IBM SPSS Statistics for Windows, Version 27.0. Armonk, NY, USA: IBM Corp) software package. All analyses were performed separately for men and women. Descriptive statistics were used for data analysis: means, standard deviations, min., max. meanings. The distributions of variables were compared in sex groups at the baseline survey using chi-square and z-tests. Mean differences were tested using a *t*-test. *p* < 0.05 values were considered statistically significant. The association between physical activity levels and mortality from IHD was investigated using multivariate Cox regression analysis and mediation analysis. Using Cox regression analysis hazard ratios (HR) were calculated with a 95% confidence interval (CI), and the model was adjusted for age, education, metabolic syndrome, smoking, assessment of psychological well-being, and dietary habits. A mediation analysis was performed using the PROCESS macro for the SPSS (SPSS, Chicago, IL, USA) statistical package (mediation analysis was performed using model 4) [15]. Process plugin allows you to assess several mediators (metabolic syndrome, smoking, and psychological well-being) in parallel at the same time and calculate the cumulative (sum) effect of mediators.

## 3. Results

The characteristics of men and women at the baseline survey of the Kaunas HAPIEE study (2006–2008) are presented in Table 2. Women were more educated, and they were more likely to have higher psychological well-being compared with men. However, the rate of metabolic syndrome and its components, such as increased waist circumference and low HDL cholesterol levels, were more prevalent in women compared with men. The rate of elevated arterial blood pressure and increased triglyceride levels were more prevalent in men compared with women; also, men were more often regular smokers, and they were more likely to have unhealthy nutrition habits compared with women. The mean number of hours of physical activity in leisure time per week was significantly higher for women compared to men. The prevalence of IHD at baseline was 21.0% among men and 22.3% among women.

During the follow-up period, there were 338 (225 men and 113 women) deaths from IHD, 848 (512 men and 336 women) deaths from other causes, and 5584 responders (2328 men and 3256 women) survived. 

Table 3 presents an association between physical activity and the risk of mortality from IHD in the Kaunas city population aged 45–72 years according to sex and IHD status at baseline. It was found that moderate and higher levels of physical activity (tertiles 2 and 3) reduced the risk of IHD mortality (HR = 0.54; *p* = 0.016 and HR = 0.60; *p* = 0.031, respectively) in men who were not diagnosed with IHD at baseline compared with physically inactive subjects (tertile 1). Also, it was found that among men who were diagnosed with IHD at baseline, higher physical activity (tertile 3) significantly reduced the risk of mortality from IHD in men compared with those who were physically inactive (HR = 0.54; *p* = 0.021), and in the women’s group, moderate physical activity (tertile 2) significantly reduced the risk of mortality from IHD compared with those who were physically inactive (HR = 0.41; *p* = 0.025).

**Figure 1:**
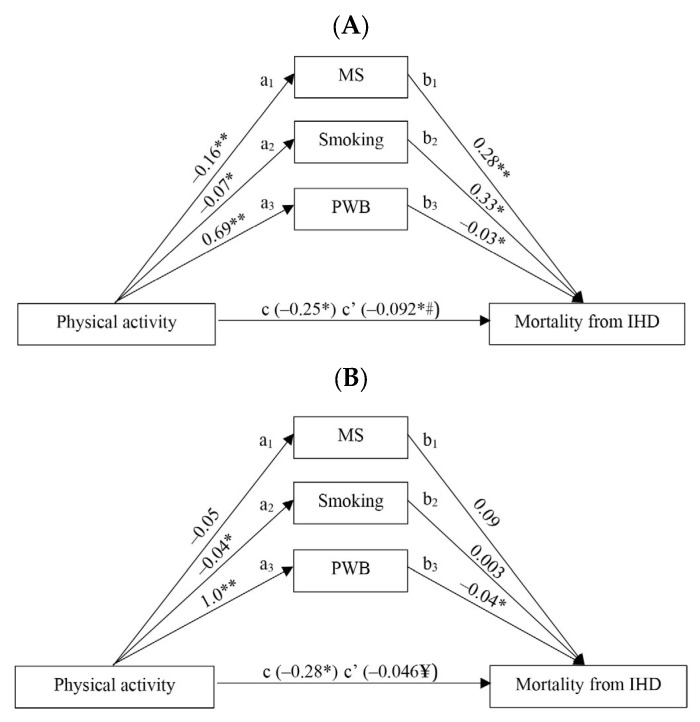
The effect of physical activity on the risk of mortality from IHD, mediated by other risk factors, in men (**A**) and women (**B**) groups aged 45–72 years. a—relationship between the input variable and the mediating variable. b—relationship between the mediating variable and the output variable. c—direct effect of an input variable on an output variable. c’—indirect effect of an input variable on an output variable. MS—metabolic syndrome. PWB—psychological well-being assessment. * *p* < 0.05; ** *p* < 0.001. # Nagelkerke R^2^ = 0.1317 for the men’s group; ¥ Nagelkerke R^2^ = 0.1952 for the women’s group.

A mediation analysis was performed to analyze the associations between physical activity and other risk factors (metabolic syndrome, smoking, and psychological well-being assessment) and mortality from IHD, controlling for age as a confounder. Figure 1 shows the effect of physical activity on mortality from IHD mediated by other risk factors in men (A) and women (B) groups aged 45–72 years. The estimation of direct associations between physical activity and the risk of mortality from IHD showed, that physical activity directly predicted a statistically lower IHD mortality risk (*p* < 0.05) in men (Figure 1A; path c) and women (Figure 1B; path c) groups. The estimation of indirect (risk factor-mediated) associations between physical activity and the risk of mortality from IHD showed that all three analyzed risk factors (metabolic syndrome, smoking, and psychological well-being) concurrently mediate the association between physical activity and mortality risk from IHD only in the men group. The more physically active men were, the less likely they were to have metabolic syndrome (Figure 1A; path a_1_) (*p* < 0.001), the less they smoked (Figure 1A; path a_2_) (*p* < 0.05), and the better they assessed their own psychological well-being (Figure 1A; path a_3_) (*p* < 0.001). As the results of those analyses showed, metabolic syndrome (Figure 1A; path b_1_) and smoking (Figure 1A; path b_2_) are associated with an increased risk of mortality of IHD, while a better assessment of psychological well-being (Figure 1A; path b_3_) is associated with a decreased risk of mortality from IHD in the men’s group (*p* < 0.05). Despite the fact, that physical activity directly reduces the risk of mortality from IHD in the women group (Figure 1B; path c), the other mediators are not so important, except for psychological well-being. Physical activity increases the psychological well-being of women (Figure 1B; path a_3_) (*p* < 0.001), which significantly reduces the risk of mortality from IHD (Figure 1B; path b_3_) (*p* < 0.05). Also, the more physically active women were, the less they smoked (Figure 1B; path a_2_) (*p* < 0.05); however, smoking was not associated with an increased risk of mortality from IHD (Figure 1B; path b_2_) (*p* > 0.05), and this was probably due to the low number of women who were smokers.

## 4. Discussion

Physical inactivity is indeed a major global public health concern. One of the reasons for the global decline in physical activity is the increasing prevalence of sedentary behavior [5,16]. This includes prolonged sitting at work, during leisure time, and while commuting. Increased use of electronic devices such as smartphones, tablets, and computers has also contributed to sedentary behavior. Research from other countries may help explain differences in leisure-time physical activity among men and women aged 45–72 in our population and why women are more physically active than men during leisure time. The systematic review analysis results about individual characteristics and physical activity in older adults showed that gender differences in physical activity levels were examined in many studies. Across all physical activity types, men were more active than women in 27 instances and less active in 7; no association between gender and physical activity level was found in 19 instances [6]. Whether or not an association between gender and physical activity level can be observed may depend on the physical activity domain. Men had higher physical activity levels than women when performing vigorous or work-related physical activity; on the other hand, women performed more physical activity in the house or garden. The research data from Australia showed that although there was overlap in motivating factors and context preferences for physical activity in women and men aged 60–67 years, there were also marked gender differences: women were more likely than men to be motivated by improving their appearance, spending time with others, meeting friends, or losing weight [7].

Our longitudinal cohort study results show that high physical activity was a significant factor that directly predicted statistically lower IHD mortality in men, regardless of whether subjects had IHD at baseline or not. However, only moderate physical activity was a significant factor that directly predicted statistically lower IHD mortality in the women’s group with IHD at baseline.

Can physical activity during leisure time really reduce the risk of dying from this disease in patients with IHD? A systematic review and meta-analysis of prospective observational studies indicated that higher levels of post-diagnosis physical activity are associated with lower mortality rates in ischemic heart disease and other non-communicable diseases, with the indication of a no-threshold and non-linear dose-response pattern [17]. On the other hand, the results from the study in Germany show that after diagnosis of an initial acute coronary syndrome, patients change their lifestyle: the proportion of patients undertaking physical exercise significantly increased, as did the consumption of fruit, vegetables, and fish, while alcohol consumption decreased [18]. Despite the fact that women with IHD may be more motivated to take care of their health and engage in physical activity due to their existing condition, the decrease in mortality from IHD among women could be determined by other factors related more to metabolic and hormonal changes occurring in the postmenopausal period than to high or moderate levels of physical activity. According to Imboden M.T. et al., observed middle-aged women >20 years who were healthy at baseline also found no significant reduction in CVD mortality when comparing high, moderate, and low levels of physical activity, but men had a significantly higher risk of CVD mortality (even up to 3 times) comparing a high level of physical activity with a low level of physical activity applying the cardiorespiratory fitness model [19]. So, the investigation of sex differences in risk factor awareness and treatment in patients with IHD is beyond the scope of this analysis but might provide a further understanding of our results and should be addressed in future studies.

Many longitudinal cohort studies have analyzed the relationship between physical activity and the risk factors of IHD, and IHD mortality risk, regardless of other chronic diseases [20,21,22]. Previous examination of physical activity in the Framingham cohort revealed that mortality due to IHD was inversely related to the level of physical activity for men [20]. The effect of being sedentary on mortality is rather modest compared to the effects of other risk factors, but in mortality due to IHD, it persists when these factors are considered; however, for women, the effect is negligible [20]. Several large-scale studies have consistently shown that individuals who engage in regular physical activity during their leisure time have a lower risk of developing and dying from IHD [21,22]. For example, a meta-analysis of 21 prospective cohort studies found that high levels of leisure-time physical activity have a beneficial effect on cardiovascular health by reducing the overall risk of incident coronary heart disease among men and women by 20 to 30% [21]. The protective effect of physical activity on IHD mortality is thought to be due to its beneficial effects on cardiovascular health, including improvements in blood lipid levels, blood pressure, glucose metabolism, and endothelial function [21,22]. Moreover, physical activity can also reduce the risk of other risk factors for IHD mortality, such as metabolic syndrome, which is a cluster of metabolic abnormalities that include central obesity, high blood pressure, high blood sugar, and abnormal blood lipid levels, all of which increase the risk of developing cardiovascular disease [23]. Physical activity can help prevent or manage metabolic syndrome by improving insulin sensitivity, reducing central obesity, and lowering blood pressure and blood lipid levels [4,23]. Similar results are also found in the studies of other scientists conducted in Europe and around the world. Moderate and intense physical activity reduced the possibility of metabolic syndrome by 3–10% for residents of the Canary Islands [24]. The Spanish population, which was less physically active during leisure time, had metabolic syndrome, was more obese, and had a larger waist circumference [25]. The results from other studies from Spain looked at an elderly population with CVD risk factors. It was found that residents with average or higher leisure time physical activity had a lower chance of acquiring metabolic syndrome [26] and participants who were physically inactive during leisure time had a higher incidence of metabolic syndrome than those who were moderately or more physically active [27]. In a cohort of American men, higher leisure-time physical activity was associated with lower odds of metabolic syndrome [28], and in a cohort of Japanese men and women with higher daily physical activity who exercised more during leisure time, the prevalence of metabolic syndrome was lower compared to those who were physically inactive [29]. It can be assumed that any physical activity in the population of any age, even for those at risk of CVD, is an important factor in avoiding metabolic syndrome. Similar results were obtained in our longitudinal study. The estimation of indirect (risk factor-mediated) associations between physical activity and the risk of mortality from IHD showed that metabolic syndrome is a significant mediator of the examined association in the men’s group.

The results of our mediation analysis indicated indirect associations between physical activity and the risk of mortality from IHD acting in combination with smoking habits, which is a significant mediator of the examined association in the men’s group. Similar results were obtained in a Norwegian study: smokers tend to be less physically active in their leisure time than non-smokers [30]. When comparing physically active Finnish residents with inactive and less active peers, it was found that the frequency of smoking was lower among increasingly active women and men and constantly active men [31]. In another population study, it was found that regularly physically active Finns are mostly non-smokers, while physically inactive and less active Finns are mostly smokers [32]. The results of this and the reviewed studies confirm that individuals who are physically active are less likely to smoke.

In addition, physical activity can improve psychological well-being by reducing symptoms of anxiety and depression, improving mood, and promoting stress management, which can indirectly reduce the risk of developing IHD [16]. The results of our mediation analysis indicated that physical activity in leisure time increases the psychological well-being of men and women, which significantly reduces the risk of mortality from IHD. The results of some studies also have shown that physical activity during leisure time can have positive effects on psychological well-being, including reduced symptoms of anxiety and depression, improved mood, and increased self-esteem [33]. It is important to note that the relationship between physical activity, psychological well-being, and IHD mortality is complex and may be bidirectional. For example, individuals who have better psychological well-being may be more likely to engage in physical activity, and physical activity may, in turn, improve psychological well-being and reduce the risk of IHD mortality. In summary, physical activity during leisure time has been associated with both improved psychological well-being and a reduced risk of IHD mortality. The exact mechanisms underlying these associations are complex and likely involve multiple factors.

There are some strengths and limitations in this study. Strengths of the present study are that it has a prospective design, a large sample size, and a wide age interval of study participants including middle-aged and elderly individuals (45–72 years at baseline). Other strengths are that data were collected using standardized and validated study methods [8], and a long follow-up period (from 2006–2008 to 2018). The novelty of this study is that various statistical methods (multivariable Cox regression analysis and mediation analysis) were used for a comprehensive assessment of the prognostic value of physical activity in leisure time on mortality from IHD in the context of other risk factors. Many potential confounders are included in statistical analyses (metabolic syndrome, smoking, assessment of psychological well-being, eating habits, age, and education). There are some limitations to our study. First, in this study, we only had the possibility to analyze how the level of physical activity affects mortality risk in IHD. However, we had no possibility to evaluate the influence of physical activity on the risk of other non-communicable diseases such as cancer and stroke for those reasons: we currently did not have more detailed data on deaths from cancer and its types; also, the percentage of deaths from stroke during the observation period was too small to perform thorough analyses. Second, we had no possibility of including in the multivariable statistical analysis the data on the treatment of IHD or the adequacy of the therapy (medication) among study participants who were diagnosed with IHD at baseline, because by study protocol, we did not collect the data about the treatment of IHD. The third limitation is that in the statistical analysis, we included some lipids (triglycerides and HDL cholesterol) which are components of the metabolic syndrome; however other lipids such as total cholesterol and low-density lipoprotein cholesterol were not included in this analysis. The fourth limitation is that confounding by other lifestyle factors such as alcohol consumption may be a plausible part of the explanation for an inverse association between physical activity and mortality risk. However, in our study, we did not adjust the data by alcohol consumption.

## 5. Conclusions

Our longitudinal cohort study suggests that engaging in regular physical activity during leisure time has been associated with a lower risk of IHD mortality, likely due to its beneficial effects on cardiovascular health and reduction of other risk factors. Preventive actions are necessary to increase physical activity and improve cardiovascular health. Reinforced efforts shall be prioritized and scaled up for broadening and ameliorating the application of physical activity guidelines, especially in specific populations with IHD or with some risk factors such as metabolic syndrome or low psychological well-being status.

## Figures and Tables

**Table 1:** Physical activity levels in population aged 45–72 years (hours/week).

Physical Activity Levels/Tertiles	Min–Max *	Mean (SD)
**MEN**		
Physically inactive (1st tertile)	0.0–10.0	5.8 (3.1)
Moderately physically active (2nd tertile)	10.5–19.5	14.7 (2.6)
Higher physically active (3rd tertile)	20.0–42.0	26.8 (5.9)
**WOMEN**		
Physically inactive (1st tertile)	0.0–13.5	8.6 (3.4)
Moderately physically active (2nd tertile)	14.0–22.0	17.5 (2.6)
Higher physically active (3rd tertile)	22.5–42.0	29.2 (5.1)
**ALL**		
Physically inactive (1st tertile)	0.0–12.0	7.2 (3.4)
Moderately physically active (2nd tertile)	12.5–20.5	16.2 (2.4)
Higher physically active (3rd tertile)	21.0–42.0	28.1 (5.7)
	22.5–42.0	29.2 (5.1)

SD—Standard deviation. * Minimum (Min) is the lowest evaluation, and maximum (max) is the highest evaluation.

**Table 2:** Baseline characteristics of men and women at the baseline survey of the Kaunas HAPIEE study (2006–2008).

Variables	MEN *n* = 3065	WOMEN *n* = 3705	*p*
**Age**, years, mean ± SD **Education**, % Secondary and lower	57.3 ± 7.87	57.1 ± 7.84	0.217
			<0.001
	46.8	37.5	
College and higher **Metabolic syndrome**, %	53.2	62.5	
	27.5	33.8	<0.001
Elevated arterial blood pressure (≥130/85 mm/Hg), % **Increased waist circumference**, % Men ≥ 102 cm, women ≥ 88 cm **HDL cholesterol**,	83.4	69.7	<0.001
		
	27.3	48.6	<0.001
		
Men < 1.0 mmol/L, women < 1.3 mmol/L, %	12.1	23.2	<0.001
**Triglycerides** ≥ 1.7 mmol/L, %	28.3	25.0	0.001
**Fasting glucose** ≥ 6.1 mmol/L, %	30.8	30.9	0.475
**Psychological well-being groups**			0.004
Higher	52.8	56.3	
Lower	47.2	43.7	
**Regular smoking**, %	37.7	13.6	<0.001
**Nutrition habits**, %			
More frequent consumption of fresh fruit and vegetables	51.1	59.4	<0.001
More frequent consumption of sweets	51.4	48.9	0.020
More frequent consumption of cereals, and infrequent consumption of meat	32.7	58.0	<0.001
More frequent consumption of meat, potatoes, and eggs	61.4	42.6	<0.001
More frequent consumption of chicken and fish	55.4	49.3	<0.001
**Physical activity** (hours/week), mean ± SD	15.3 ± 9.39	18.2 ± 8.96	0.024
**Prevalence of IHD at baseline survey**, %	21.0	22.3	0.197

Data weighted by age (WEIGHT10). The chi-square test for distributions and the T-test for means and the z-test for two percentages were used to compare differences in variables between sexes. SD—standard deviation; HDL—high-density lipoprotein; IHD—ischaemic heart disease.

**Table 3:** An association of physical activity with the risk of mortality from IHD in Kaunas city population aged 45–72 years according to sex and IHD status at baseline study.

	IHD Status			
	Without IHD HR (95 % CI)	*p*	With IHD HR (95 % CI)	*p*
**MEN**	*n* = 2422		*n* = 643	
Physically inactive	1		1	
Moderately physically active	0.54 (0.33–0.89)	0.016	0.69 (0.43–1.10)	0.121
Higher physically active	0.60 (0.37–0.95)	0.031	0.54 (0.32–0.91)	0.021
**WOMEN**	*n* = 2877		*n* = 828	
Physically inactive	1		1	
Moderately physically active	0.75 (0.40–1.39)	0.354	0.41 (0.19–0.89)	0.025
Higher physically active	0.73 (0.38–1.38)	0.331	0.54 (0.25–1.18)	0.123
**ALL**	*n* = 5299		*n* = 1471	
Physically inactive	1		1	
Moderately physically active	0.63 (0.43–0.92)	0.017	0.54 (0.37–0.81)	0.003
Higher physically active	0.56 (0.39–0.82)	0.003	0.48 (0.31–0.74)	<0.001

IHD—ischaemic heart disease. HR—hazard ratio. CI—confidence interval. Multivariate Cox regression. Variables included in the model: metabolic syndrome, smoking, assessment of psychological well-being, eating habits, age, and education. Reference group: physically inactive—tertile 1. Moderately physically active—tertile 2. Higher physically active—tertile 3.

## Data Availability

Research data are available by request to the corresponding author.
